# Electric Field Intensity Effects on the Microstructural Characteristics Evolution of Methyl Vinyl Silicone Rubber via Molecular Simulation

**DOI:** 10.3390/molecules23081861

**Published:** 2018-07-26

**Authors:** Ying Liang, Ting Gao, Xiangnian Wang, Mengting Sun, Lijuan Gao

**Affiliations:** 1 Hebei Provincial Key Laboratory of Power Transmission Equipment Security Defense, North China Electric Power University, Baoding 071003, Hebei, China; lylxj527@126.com (Y.L.); wang_xiang_nian@sina.com (X.W.); smtkkll@126.com (M.S.); 2 Hengshui Power Supply Branch, State Grid Hebei Electric Power Supply Co., Ltd., Hengshui 053000, Hebei, China; ncepuglj@163.com

**Keywords:** composite insulator, methyl vinyl silicone rubber, molecular dynamics, electric field, elastic modulus

## Abstract

During ultra high voltage (UHV) transmission, the discharge caused by high intensity electric fields aggravates the aging process of external insulation materials used for composite insulators. The microstructural characteristics of its base material polymer—methyl vinyl silicone rubber—are the key basis for the performance of insulation materials under electric field exposure. Based on molecular dynamics simulations, a molecular model of methyl vinyl silicone rubber was established. Mechanisms influencing the microstructure evolution under electric fields had been studied at the atomic level. The results showed that the initial reaction characteristics of silicone rubber molecules involve the violent vibrated of all the methyl and vinyl atoms, and shortening of the chemical bonds. The neighboring groups were close to each other and generated different amounts of -Si-Si- bonds. This promoted the helical shrinkage of the molecule, and protrusion of the middle of the molecule which presented an inverted U shape. The high electric field greatly reduced the total energy of molecules, and the potential energy in particular was more severely destroyed, resulting in degradation of its structure. Besides, as the electric field intensity increased, the elastic modulus of the molecule gradually increased. It was shown that high electric fields would make the stiffness of silicone rubber become larger, and the brittleness to become stronger, which reduced the mechanical properties of materials, accelerating its aging. The results provide a theoretical basis for establishing the connection between the micro appearance and macro characteristics of materials, as well as reference values for the optimization of base materials used for making composite insulators.

## 1. Introduction

As composite insulators have excellent performance, they have obvious advantages in the external insulation selection for ultra high voltage UHV transmission projects [1]. However, the surface of composite insulators in UHV projects will face strong electric field intensities. Because of its longer insulation distances, the field intensity of the high voltage side is more concentrated [2]. Continuously concentrated high electric fields cause serious damage to the electrical and mechanical properties of composite insulators, which can deform or even crack the polymer structure of silicone rubber [3], and as a result its chemical structure, hardness, elasticity, and other physicochemical properties are subject to change [4]. It can be seen that in the high electric field environment of UHV, aging problems of composite insulators should not be underestimated.

In most cases researchers have studied composite insulators aged by electric field experiments. Reference [5] shows that a high DC voltage field will break the chemical bonds of insulator surfaces and further affect their surface hydrophobicity. Liang et al. at Tsinghua University [6,7,8] believe that the chemical structure changes of silicone rubber aged by corona discharges are the direct cause of its performance changes. Reference [9] shows that the trap density of silicone rubber increases significantly with prolongation of corona aging time, and many chemical groups on the surface also change. Tu et al. [10] performed electrical aging simulations on composite insulators and found that, the higher the electric field intensity, the worse the hydrophobicity on the surface.

The current studies are mainly focused on discharge aging of insulation materials induced by increasing electric fields. The aging process of materials ranges from quantitative changes to qualitative changes. However, the above studies rarely examined the structural evolution of the material as the electric field intensity increases. When the material is initially placed in an increasing electric field, changing characteristics of internal structure are the early stage of discharge aging and further degradation. The above studies derived and analyzed the aging state of materials from macroscopic characterizations, which are the cumulative result of the material’s micro characteristics, but the changes of the micro characteristics are difficult to observe directly from experiments.

Due to the limitations of experimental means, the molecular simulation method has attracted more attention and has been widely used, especially in the micro characteristics of the material field, where several remarkable achievements have been made. For example, the simulation method has been applied in the cellulose degradation process of oil paper insulation by Liao et al. [11,12]. Via this study they explored the diffusion behavior of moisture and acid in the insulation paper during follow-up work. Chinese professors Wang [13] and Li and Zhang [14,15,16] et al. performed molecular simulations of cellulose under different temperatures for analyzing the pyrolysis mechanism of insulation paper. Based on high efficiency of Reax FF reaction [17], the high temperature cracking process of insulation paper was simulated by literature [18]. The initial pyrolysis stage of phenolic resin was simulated by [19] at different temperatures. Besides, Paavilainen et al. [20] and references [21,22] investigated the influence of temperature on hydrogen bonds and related properties of molecular chains in cellulose structures. Reference [23] studied the melting process of *β*-cristobalite under different electric fields. The influence factors of polymer dynamic stability were studied in [24,25]. The density functional calculation model of cellulose was explored in depth in [26]. It is evident that the macroscopic characteristics and basic rules of systems can be obtained by molecular dynamics method to further study movement laws of micromolecules [27].

The most used base polymer for composite insulators at home and abroad is methyl vinyl silicone rubber (MVQ) [28]. Based on the molecular dynamics theory, a molecular model of MVQ was established. The evolution process of silicone rubber microstructure under different electric field intensities was explored. The changes of related characteristics and chemical bonds were statistically analyzed. Then the evolution laws of the mechanical properties were analyzed in depth. From the atomic level, the initial reaction mechanism of silicone rubber in an electric field has been revealed.

## 2. Parameter Calculation and Basic Model

### 2.1. Parameter Calculation

As the electric field plays a key role on the properties of silicone rubber, electrostatic interaction is calculated by the Ewald summation method. In the method, each particle interacts with the mirror image of all other particles in the simulation box and all the particles in the surrounding lattice. It is also assumed that each particle is surrounded by the neutralized charge distribution with equal opposite sign charge. Thus, the electrostatic interaction is the sum of the interaction between Gaussian functions (*E*_Gauss_), the real space (*E*_real-space_), the reciprocal space (*E*_recip-space_) and the corrected energy term (*E*_corr_)of the Gaussian distribution [29], as indicated by Equation (1):

(1) $$\begin{array}{l} E=E_{\mathrm{real}-\mathrm{space}}+E_{\mathrm{recip}-\mathrm{space}}+E_{\mathrm{Gauss}}+E_{\mathrm{corr}}\\ =\frac{1}{2}\sum_{i=1}^{N}\sum_{j=1}^{N}\{\begin{array}{c} +\sum_{n=0}^{\infty }\frac{q_{i}q_{j}\mathrm{erfc}\left(\alpha \left|r_{\mathrm{ij}}+n\right|\right)}{\left|r_{ij}+n\right|}\\ +\sum_{k\neq 0}\frac{4\pi q_{i}q_{j}}{k^{2}L^{3}}\exp\left(-\frac{k^{2}}{4\alpha^{2}}\right)\cos\left(k\cdot r_{\mathrm{ij}}\right)\\ -\frac{\alpha }{\sqrt{\pi }}\sum_{k=1}^{N}q_{k}^{2}+\frac{2\pi }{3L^{3}}{\left|\sum_{k=1}^{N}q_{k}r_{k}\right|}^{2} \end{array} \end{array},$$

In the formula, *N* is the charged particles, *q* is charge quantity, *r* is particle distance, *L* is side length of simulation box, *k* = 0, 1, 2, …, *α* is a constant and its usual amount of calculation is on the order of *N*^2^, *n* is the coordinates of a mirrored box. 

The basic characteristics of the molecular chain, such as potential energy, kinetic energy, kinetic constants and bond lengths were calculated. In order to explore the relationship with macroscopic characteristics of silicone rubber, the elastic modulus parameters were also calculated. Bulk modulus, shear modulus and Young’s modulus are important indicators for reflecting the ability of materials to resist elastic deformation. The larger the values, the greater the stiffness. Values are also related to the chemical structure, composition, microstructure and temperature of the materials. Based on the Young’s modulus and shear modulus values obtained from simulations, and according to the derivation of Hooke’s law, the elastic constant and bulk modulus were calculated in combination with Equations (2) and (3):

(2) E=2G(1+ν),

(3) $$K=\frac{E}{3\left(1-2\nu \right)},$$

In the formulas, *E* is the elastic constant, *G* is shear modulus, *K* is bulk modulus, and *v* is Poisson’s ratio.

### 2.2. Molecular Structure

**Figure 1:**
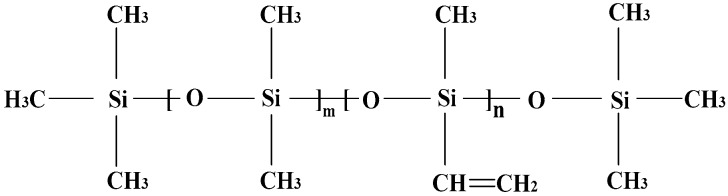
Molecular structure of polymethylvinyl siloxane.

The main material of MVQ is polymethylvinyl siloxane, which is a high molecular copolymer of dimethyl siloxane and methylvinyl siloxane chain segments. The molecular structure can be seen in Figure 1. The molecular chain is made of -Si-O-Si- bridging bonds, while Si atoms are mainly linked to -CH_3_, and a small amount of unsaturated groups are introduced on side chains, such as -CH=CH_2_. The intermolecular forces are small, and methyl groups arranged outwards can rotate freely, so the silicone rubber has a certain degree of hydrophobicity.

### 2.3. Molecular Model and Optimizations

**Figure 2:**
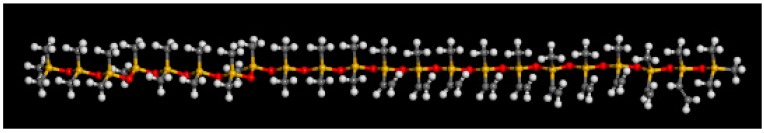
Initial molecular model. O: red, Si: yellow, C: gray, H: white.

The molecular weight Mr of MVQ is: (4.0~6.0) × 10^5^. It is impractical to model 400~800 thousand molecules and carry out simulations under different electric field intensities. However, the polymerization degree was too small to represent the basic characteristics of the polymer. Some scholars have proposed that there are coarse-grained models allowing simulations of long polymer chains [30,31]. In order to carefully explore the changes of atomic bond length and motion, etc. a molecular model of MVQ silicone rubber containing 237 atoms with a polymerization degree of 10 was established, as shown in Figure 2. 

**Figure 3:**
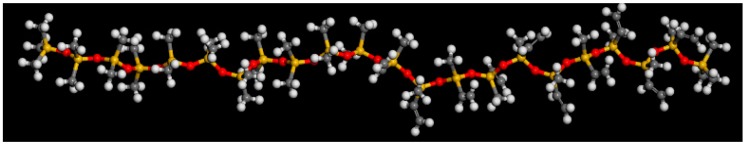
Optimized molecular structure.

To simulate the molecule evolution at room temperature, the field was set to 298 K. Moreover, the model didn’t take other fillers or impurities into account. Based on the Materials Studio software the Forcite/COMPASS force field was used to conduct optimizations of molecular geometry and energy minimization. The COMPASS force field incorporates many ab initio calculations of quantum mechanics and has many parameters for metals and metal oxides. Moreover, it is the first high quality molecular force field that unites organic and inorganic molecular parameters in the same force field. Its correctness has been verified by a total of 28 molecules of single molecules, liquid molecules and crystal molecules [11]. The 20,000 steps were selected for geometry optimization, and 5000 steps were used for the optimization of energy minimization. The time step is 1 fs. The purpose of optimizing the molecule is to obtain a more stable molecular structure. The calculated method of electrostatic is Ewald, van der Waals is atom based. NVT ensemble is selected to obtain the required environment of simulations. Its effectiveness can be proved by [11,12,13,19,20]. After optimizations, the molecular chain shrinks inward and presents a curved shape, as shown in Figure 3. The box size of the chain is 13.5 Å × 13.5 Å × 13.5 Å with the cutoff distance of 6.5 Å. In order to conveniently label changes of the molecular chain, the box of Figure 3 was removed.

As can be seen from Table 1, molecular energies of silicone rubber change significantly after optimizations. Where total potential energy, bond energy, van der Waals energy and electrostatic energy, etc. decrease so greatly that the current molecular structure is considered to be stable for further calculation. Therefore, based on the Ewald summation method, part of the atomic charges are shown in Table 2.

### 2.4. Calculation of Molecular Force Field

Considering research objects contains C, H, O, Si atoms, the Dreiding force field was selected to calculate the structure and properties of molecular aggregates, so MD calculations were carried out at different electric field intensity by the GULP/Dreiding package. The calculations were run for 5000 steps along with a 1 fs time step on the well at a temperature of 298 K. The relaxation time is 1 ps. The electric field was set in the X direction from 0.001 V/Å to 3 V/Å with an interval of 0.2 V/Å and two additional fields 0.01 V/Å and 0.1 V/Å.

## 3. Results and Discussion

### 3.1. Calculation of Molecular Force Field

**Figure 4:**
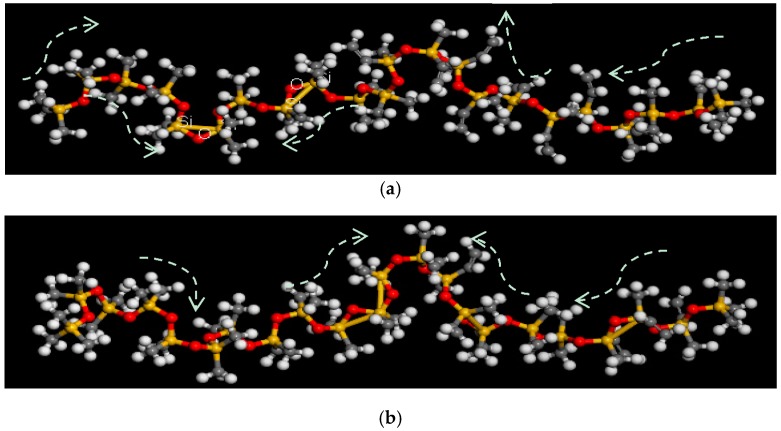

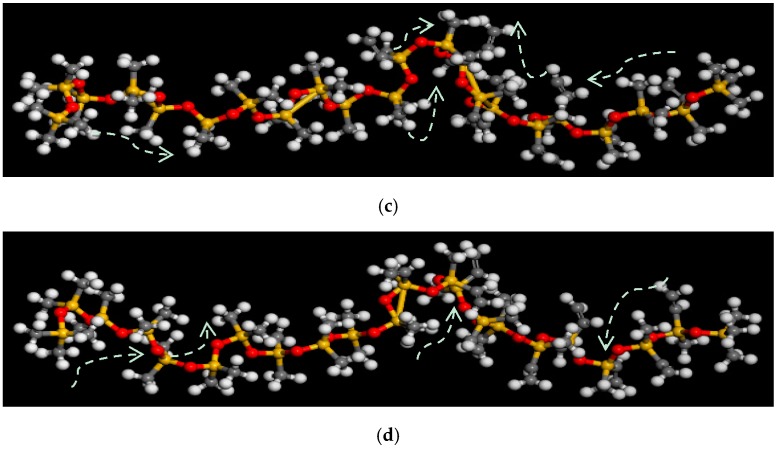
Structure changes of molecule chains during different electric fields. (**a**) The molecule in the 0.001 V/Å electric field; (**b**) The molecule in the 0.01 V/Å electric field; (**c**) The molecule in the 0.1 V/Å electric field; (**d**) The molecule in the 1 V/Å electric field.

Figure 4 shows the molecular chain changes under different electric fields, in which the directions of white arrows represent contraction trends in the molecule. One can notice that the molecular chain of silicone rubber shrinks from the two ends to the middle, resulting in a helix central protrusion and forming an inverted U shape. The methyl and vinyl groups attached to Si atoms vibrate vigorously while both -Si-C- and -Si-O- bonds accordingly rotate. -Si-Si- bonds are formed on the basis of -Si-O-Si- bonds to form a triangular structure, while there is a simultaneously explosive change in bond lengths and bond angles of -CH_3_ as well as -CH=CH_2_. As the electric field intensity increases, there is a significantly shrinked molecule with a more obvious helical structure. Meanwhile, group^,^ rotations are larger and the number of -Si-Si- bonds constantly changes. There is a tendency for -Si-O-Si- bridging bonds to form a ring structure with shortened -C-H bonds and the molecular length is significantly reduced.

### 3.2. Molecular Energy Changes

**Figure 5:**
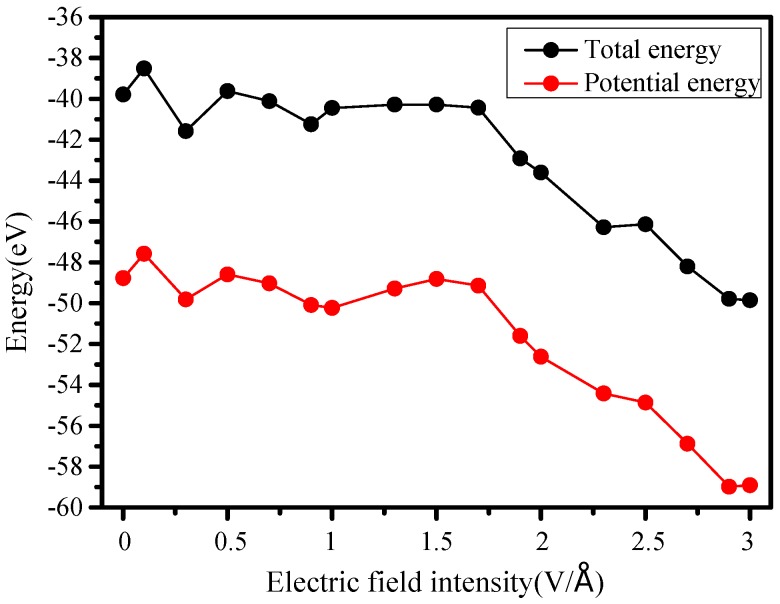
Changes of total and potential energy constant.

By statistical analysis, obtained trends of total energy and potential energy in each run are shown in Figure 5. When the electric field intensity is less than 1.7 V/Å, energies show a gradual downward trend due to continuously increasing electric fields, but its change trend was relatively stable, which can be considered as a transitional period. After the field reaches 1.7 V/Å, as a result of decreasing total energy and potential energy, the damage of electric field force on the molecule is prominent.

**Figure 6:**
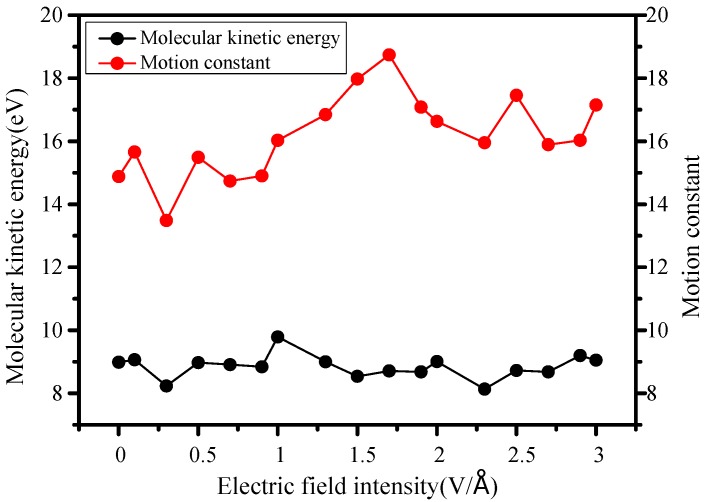
Changes of kinetic energy and motion.

At the same time, as can be seen from Figure 6, the system is mainly embodied in intense movement of atoms, the molecule do not vary much in size except for contraction, so the motion constant increases slightly to reach the maximum at 1.7 V/Å, followed then by another continuous increase stage. Besides, it also can obtain from Figure 5, originating from a stable temperature field with 298 K, kinetic energy remains basically constant, so its variation is relatively small.

The potential energy is a part of total energy, and its change trend is basically the same as total energy (Figure 5), which indicates that the decrease of total energy is mainly caused by potential energy changes. With changing laws of molecular structure in Section 3.1, it is known that high electric field stress will obviously destroy the molecular potential energy in a weak temperature field (298 K), resulting in spiral contraction and degradation of the structure.

### 3.3. Internal Temperature Changes

**Figure 7:**
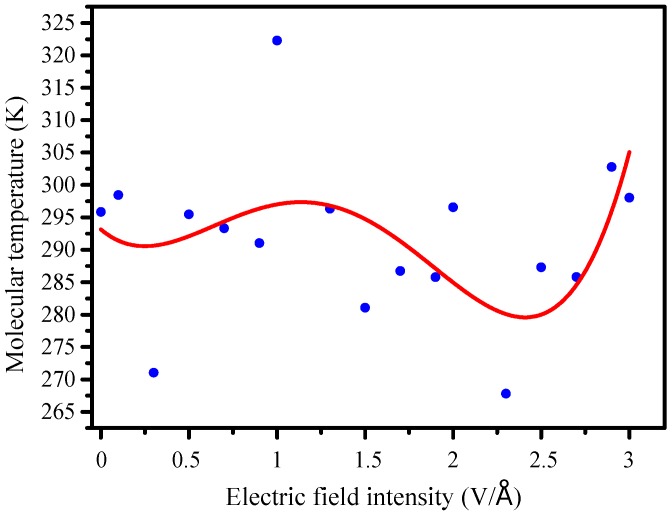
Changes of molecular internal temperature.

It can be found in Figure 7, internal temperature of the molecule slowly rises and drops, then increases again under rising electric field intensity, but the motion constant increases gradually when there is less than 1.5 V/Å (Figure 6), the atomic vibration increases and internal temperature rises. When the electric field further increases over 1.5 V/Å, the higher internal temperature of early stage causes molecular energy losses, weakening its atomic vibrations, so the internal temperature decreases.

The molecular structure has undergone a significant degradation with greatly reduced potential energy under further increased intensity. There was no doubt that the molecules’ ability to resist an external electric field is greatly weakened, the internal temperature swinging increases, leading to further destruction of molecular potential energy.

### 3.4. Number of -Si-Si- Changes

**Figure 8:**
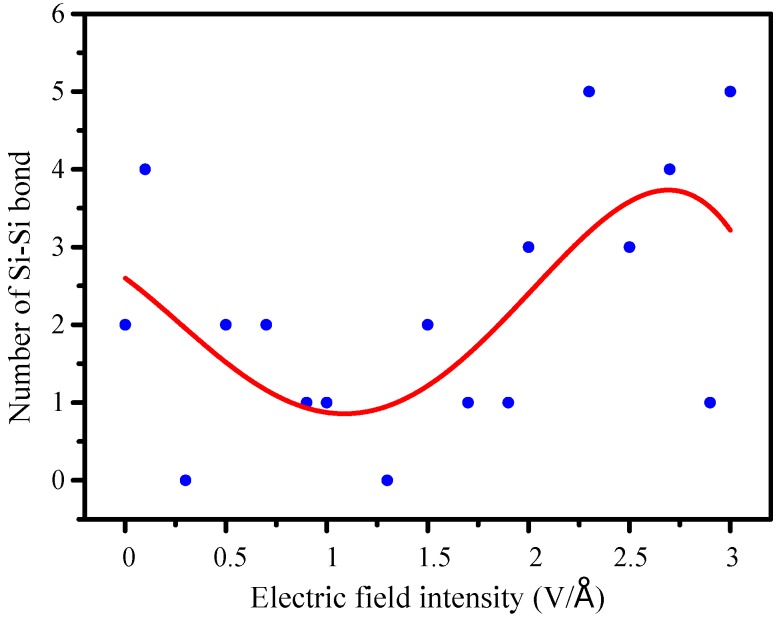
The number of -Si-Si- bond.

Because the molecular chain shrinks helically, making two Si atoms on the -Si-O-Si- bond close to each other, so Si-Si bonds are generated by interatomic van der Waals force and their own changed energy. An unequal number of -Si-Si- bonds which present a scatter point distribution at various electric field intensity is shown in Figure 8.

To explore its distribution pattern, a curve fitting (the red curve in the Figure 8) is carried on the distribution graph. The number of bonds exhibits a function of a quartic polynomial regression with electric field intensity, the relationship is shown in the Equation (4):

(4) $$Y=A+B_{1}X+B_{2}X^{2}+B_{3}X^{3}+B_{4}X^{4},$$

In the lower electric field intensity, initial movements of atoms are more intense, so it is difficult to generate -Si-Si- bonds. Therefore, when the field is less than 1.3 V/Å, the number of -Si-Si- bonds gradually decreases with rising field intensity. Along with the further increasing intensity, the internal temperature is gradually reduced, the molecular energy decreases more sharply, the strength of atomic movement slows down and it is easy to generate -Si-Si- bonds. Moreover, as the number of bonds increases, helical shrinkage of molecular structure becomes more obvious. It can be seen that the generation of -Si-Si- bonds is a necessary stage during the process of molecular structural degradation, which will promote further degradation of molecules.

### 3.5. Chemical Bonds Changes

**Figure 9:**
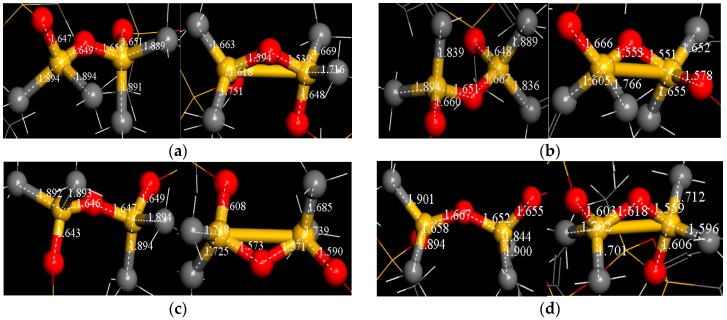
Bond lengths under different electric fields. (**a**) Bond lengths of Si74-Si3 in 0V/Å (left) and 0.001 V/Å (right) electric fields; (**b**) Bond lengths of Si117-Si106 in 0 V/Å (left) and 0.01 V/Å (right) electric fields; (**c**) Bond lengths of Si3-Si1 in 0 V/Å (left) and 0.1 V/Å (right) electric fields; (**d**) Bond lengths of Si228-Si196 in 0 V/Å (left) and 1 V/Å electric fields.

As in the original paper [32], bond length is one of the most important parameters for characterizing the molecular structure. In order to further explore causes of molecular structure changes and Si-Si bonds formation, some molecular chains under electric field intensities of 0.001 V/Å, 0.01 V/Å, 0.1 V/Å and 1 V/Å are chose. The bonds Si74-Si3, Si117-Si106, Si3-Si1, Si228-Si196 with representativeness are selected for examples. Then length changes of corresponding Si-O bonds and Si-C bonds are statistically analysed. The relationships between connected bonds and main chemical bonds are shown in Figure 9 as well as bond lengths.

Taking bonds Si74-Si3 and Si228-Si196 as examples, bond lengths are shown in Table 3. Statistically, it is found that all Si-C and Si-O bonds are obviously shortened, lengths of Si-C bonds vary greatly, while Si-O lengths have relatively small changes. Other bonds have the same phenomenon.

Under field stresses, the structure of silicone rubber molecules changed towards the evolution of energy minimization. Chemical bonds would shorten and release energy to a new stable state. As paper [27] mentions, for generating new bonds, it needs to overcome the effect of electric field stress and electronegativities of neighboring groups by continuously absorbing energy. According to the above paper, Si-C bonds and Si-O-Si bridging bonds release energy when bonds are shortened. Simultaneously, the distances between two neighboring Si atoms are also continuously shortened. Atoms gradually close to each other by field stresses and their attractive interactions, so the molecule appears a shrinkage phenomenon.

Besides, since Si-O bond energy is 460 kJ/mol, Si-C bond energy is 347 kJ/mol and compared with Si-Si bond of 176 kJ/mol, the former two have larger bond energy while the latter is smaller, but the energy of the latter is high, the heat that needs to be absorbed during changing process is low [33], so Si-Si bond is easy to generate.

From the above, it can be seen that the energy released by shortened Si-O and Si-C bonds mostly contributes to the formation of Si-Si bonds under electric field stresses. Furthermore, one part energy of the molecule is consumed and dissipated by atomic heat movements, while the other part is used to overcome van der Waals between atoms for generating new bonds. The whole system maintains the dynamic balance of energy at any moment.

It should be noted that, due to the lack of equivalence of simulation time and the actual on-site aging time [11], under electric field stresses of a short duration, bond energies of Si-O, Si-C and C-H are so larger that three kinds of bonds have no obvious fracture phenomena. 

### 3.6. Elastic Modulus Changes

**Figure 10:**
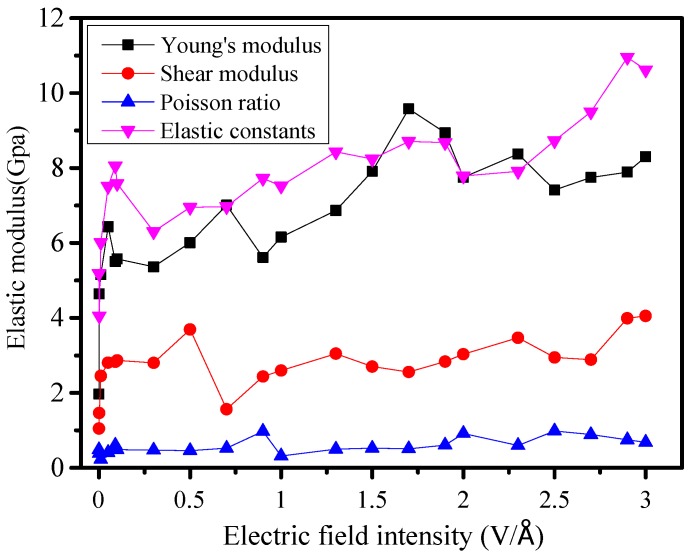
Changes of elastic modulus.

Young’s modulus can characterize the ability of the material to resist deformation. Shear modulus is the ability to characterize the materials’ resistance to shear strain. Poisson ratio is a spring constant with reflecting lateral deformation of materials. Based on main mechanical parameters simulated under different electric fields, elastic constants is calculated. The results are shown in Figure 10.

With the increase of electric field intensity, the elastic constant has the most obvious rising trend. Although Young’s modulus fluctuates greatly, it also shows an upward trend. Poisson’s ratio and shear modulus increase less with more stable trends. The gradually increasing elastic modulus shows that the ability of silicone rubber for resisting deformation increases, that is, the deformation under a certain stress is smaller, which means that the larger electric field makes rigidity of the material increase, the brittleness increases, and aging process is also accelerated. This phenomenon is consistent with causes of aging cracks on the surface of composite insulators during actual operation.

## 4. Conclusions

Based on molecular simulations, the effects of electric field on the microstructure evolution of silicone rubber molecule were studied. The results can establish theoretical basis for the relationship between the micro morphology and macroscopic characteristics of the materials after further aging and degradation. Furthermore, it can provide references for optimizations of base materials used for composite insulators. What results have been found are as follows:

(1) Under a constant electric field, the initial reaction of silicone rubber molecule is the atoms of methyl and ethylene groups vibrate violently, chemical bonds are shortened, and neighboring groups are close to each other. During the process, an unequal number of -Si-Si- bonds are generated, which promotes spiraling shrinkage of the molecule. Then the middle protruding of molecular chain presents an inverted U shape.
(2) Higher electric field intensity will cause a significant decrease in the total energy of the silicone rubber molecule, especially destruction of molecular potential energy, which results in the degradation of molecular structure. The continuous high electric field intensity will reduce molecular energies and provide a basis for further degradation of the materials.
(3) As the electric field intensity increases, the elastic modulus of molecular chain gradually increases. It shows that strong electric field will increase the rigidity of the materials, and the brittleness will become stronger, which will reduce mechanical properties of the materials and accelerate its aging process.

## Figures and Tables

**Table 1:** Molecular energies before and after optimization.

Energy (kcal/mol)	Pre-Optimization	Optimized
total potential energy	649.891	−1817.707
bond angle bending energy	319.320	88.625
dihedral angle torsion energy	−68.015	−55.556
bond length telescopic energy	993.134	3.385
non-bond interaction energy	−416.838	−1842.585
van der Waals energy	1162.035	−52.598
electrostatic energy	−1578.873	−1789.987

**Table 2:** Partial atomic charges.

Atomic Label	Charge(e)
Si1	0.71500
O1	−0.44500
C1	−0.29400
H1	0.05300
Si1	0.62750
Si1	0.71500
Si1	0.80700
C2	−0.35380
C2	−0.25360
H1	0.12680

**Table 3:** Changes of bond lengths.

Bond		Bond Length without Electric Field (Å)	Bond Length with Electric Field (Å)	Variation of Bond Length (Å)
Si74-Si3 (0.001 V/Å)	Si3-C6	1.894	1.663	0.231
	Si3-C7	1.894	1.751	0.143
	Si3-O73	1.649	1.594	0.055
	Si3-O2	1.647	1.618	0.029
	Si74-C78	1.889	1.716	0.173
	Si74-C77	1.891	1.669	0.222
	O73-Si74	1.656	1.539	0.117
	Si74-O75	1.651	1.648	0.003
Si228-Si196 (1 V/Å)	Si228-C229	1.894	1.701	0.193
	Si228-C230	1.901	1.702	0.199
	Si228-O237	1.667	1.618	0.049
	Si228-O227	1.658	1.603	0.055
	Si196-C198	1.844	1.596	0.248
	Si196-C197	1.900	1.712	0.188
	O237-Si196	1.652	1.559	0.093
	Si196-O206	1.655	1.606	0.049
